# Consumption of Fruit or Fiber-Fruit Decreases the Risk of Cardiovascular Disease in a Mediterranean Young Cohort

**DOI:** 10.3390/nu9030295

**Published:** 2017-03-17

**Authors:** Pilar Buil-Cosiales, Miguel Angel Martinez-Gonzalez, Miguel Ruiz-Canela, Javier Díez-Espino, Ana García-Arellano, Estefania Toledo

**Affiliations:** 1 Atención Primaria, Servicio Navarro de Salud-Osasunbidea, 08010 Navarra, Spain; pilarbuil@ono.com (P.B.-C.); javierdiezesp@ono.com (J.D.-E.); 2 Centro de Investigación Biomédica en Red Fisiopatología de la Obesidad y Nutrición (CIBERobn), Instituto de Salud Carlos III, 28029 Madrid, Spain; mamartinez@unav.es (M.A.M.-G.); mcanela@unav.es (M.R.C.); 3 IdiSNA, Navarra Institute for Health Research, 31008 Pamplona, Navarra, Spain; 4 Department of Preventive Medicine and Public Health, University of Navarra, 31008 Pamplona, Navarra, Spain; 5 Department of Nutrition, Harvard School of Public Health, Boston, MA 02115, USA; 6 Department of Emergency, Complejo Hospitalario de Navarra Servicio Navarro de Salud-Osasunbidea, 31008 Pamplona, Navarra, Spain; agarare@gmail.com

**Keywords:** fiber, fruit, vegetables, cardiovascular disease, legumes, whole grains

## Abstract

Fiber and fiber-rich foods have been inversely associated with cardiovascular disease (CVD), but the evidence is scarce in young and Mediterranean cohorts. We used Cox regression models to assess the association between quintiles of total fiber and fiber from different sources, and the risk of CVD adjusted for the principal confounding factors in a Mediterranean cohort of young adults, the SUN (Seguimiento Universidad de Navarra, Follow-up) cohort. After a median follow-up of 10.3 years, we observed 112 cases of CVD among 17,007 participants (61% female, mean age 38 years). We observed an inverse association between fiber intake and CVD events (*p* for trend = 0.024) and also between the highest quintile of fruit consumption (hazard ratio (HR) 0.51, 95% confidence interval (CI) 0.27–0.95) or whole grains consumption (HR 0.43 95% CI 0.20–0.93) and CVD compared to the lowest quintile, and also a HR of 0.58 (95% CI 0.37–0.90) for the participants who ate at least 175 g/day of fruit. Only the participants in the highest quintile of fruit-derived fiber intake had a significantly lower risk of CVD (HR 0.52, 95% CI 0.28–0.97). The participants who ate at least one serving per week of cruciferous vegetables had a lower risk than those who did not (HR 0.52, 95% CI 0.30–0.89). In conclusion, high fruit consumption, whole grain consumption, or consumption of at least one serving/week of cruciferous vegetables may be protective against CVD in young Mediterranean populations.

## 1. Introduction

Cardiovascular disease (CVD) is the leading cause of morbidity and mortality in developed countries [1,2] and its prevalence is increasing in developing countries. Lifestyles are the most important determinants of CVD, among which nutritional habits play an important role.

Higher dietary fiber intake has been recognized to be inversely associated with coronary heart disease (CHD) [3] and stroke [4]. Nevertheless, studies that have assessed the association between fiber intake and CVD in Mediterranean countries are scarce. It is also noteworthy that the foods that mainly contribute to the high intake of fiber—namely fruits, vegetables, legumes, and whole grains—have other micronutrients that have been postulated to be even more important for the prevention of CVD [5,6].

Fruits and vegetables are a heterogeneous food group with different contents of vitamins, minerals, and other bioactive phytochemicals [7]. There are not many studies that have evaluated the association between specific fruits or vegetables or groups of them, and it is unclear which fruit or vegetable subgroup may be the most protective against CVD. Also, fruits and vegetables consumed in different regions differ, and this could explain the differences observed in their reported effects [8].

Epidemiologic studies that have analyzed the relationship between fruit and vegetable consumption, which represent the principal source of fiber in Mediterranean countries, and CHD found inconsistent results. A meta-analysis published in 2015 [8], that analyzed 23 studies assessing this relationship, found an inverse association but showed heterogeneity with geographical differences. Concretely, an inverse association between fruit and vegetable consumption and CHD was observed only in Western countries. In addition, only one of the included studies had been conducted in a Mediterranean country [9] and this latter study found no significant association of fruit and vegetable consumption with the risk of CHD. More recently, two studies [10,11] conducted within the PREDIMED trial (PREvención con DIeta MEDiterránea) that recruited older participants in the Mediterranean area found an inverse association between fruit consumption and cardiovascular mortality, but not with non-fatal CVD. Albeit there are cohort studies and even a meta-analysis [12] that have observed an inverse association between fruits and vegetables and stroke, none of the included studies had been conducted in Mediterranean countries.

On the other hand, legumes and whole grains are also good sources of dietary fiber, and both have been suggested to be protective against CVD [13,14,15]. There is a wide body of evidence regarding the inverse association between whole grains and the risk of cardiovascular risk factors, such as type 2 diabetes [16,17], dyslipidaemia [18,19], high blood pressure [20], and obesity [17], but the research on their association with CVD clinical hard end-points is not large. A meta-analysis [21] included only nine publications that assessed the relationship between whole grains and CVD and found an inverse association. Six out of the nine studies had been conducted in the US, and only one in a Mediterranean country, and this latter study only reported on cardiovascular mortality. Even less are the studies which have assessed the relationship between legume consumption and the risk of CVD events [19,22,23].

Additionally, most of the studies have been conducted in aged adults and older people, and none in young adults. Therefore, we analyzed the association between fiber-rich foods and CVD in a Mediterranean cohort of young adults in order to advance in the knowledge of this relationship.

## 2. Materials and Methods

The SUN (Seguimiento Universidad de Navarra, Follow-up) project is a prospective, multipurpose, dynamic cohort of university graduates conducted in Spain. As a dynamic cohort, the recruitment of the participants is permanently open. Methodological aspects of this cohort have been published in detail previously [24]. Briefly, the cohort began in December 1999 and the recruitment of participants is permanently open. Information is gathered biennially by mailed questionnaires. Several validation studies of this self-reported information have been conducted, including anthropometric data [25], physical activity [26], the diagnosis of hypertension [27] and the specific criteria used for metabolic syndrome definition [28], all of them supporting the quality of the collected information.

For the present study, we included 22,476 subjects recruited before March 2013, so that they could have at least one follow-up questionnaire by the time of the last update of the dataset (December 2015). For the current analysis, we excluded 2113 participants with an energy intake out of sex specific predefined limits (>4000 kcal/day and <800 kcal/day for men and >3500 kcal/day and <500 kcal/day for women) [29]. We also excluded 947 participants who had prevalent cardiovascular disease at baseline and 2409 subjects with no follow-up information. After exclusions, the final sample population included a total of 17,007 participants. The retention in the cohort was 88%.

The study was approved by the Institutional Review Board of the University of Navarra on 30 August 2001. Voluntary completion of the first questionnaire was considered to imply informed consent.

Dietary intake was assessed through a validated 136-item semi-quantitative food-frequency questionnaire [30,31], which showed an intra-class correlation coefficient of 0.71 for fruits and 0.82 for vegetables.

Each item in the questionnaire included a typical portion size. Daily food consumption was estimated by multiplying the portion size by the consumption frequency for each item (nine options ranging from never or almost never to six times per day). Nutrient intake was estimated using Spanish food composition tables. We defined moderate alcohol consumption between 5 and 25 g/day for women and between 10 and 50 g/day for men. Based on the dietary information collected with the food-frequency questionnaire we calculated a score of adherence to the traditional Mediterranean diet [13].

Incidence of cardiovascular events, defined as non-fatal myocardial infarction, non-fatal stroke reported by participants on a follow-up questionnaire, or deaths due to cardiovascular disease, was the primary endpoint. An expert panel of physicians, blinded to the information on diet, anthropometric indexes, and risk factors, reviewed medical records of participants and adjudicated events applying universal criteria for myocardial infarction and clinical criteria for the other outcomes. A non-fatal stroke was defined as a focal neurological deficit of sudden onset and vascular mechanism lasting >24 h. Cases of fatal stroke were documented if there was evidence of a cerebrovascular mechanism. Deaths were reported to our research team by the participants’ next of kin, work associates, and postal authorities. For participants lost to follow-up, we consulted the National Death Index every six months to identify deceased cohort members and to obtain their cause of death. Cases of fatal CHD or stroke reported by families or postal authorities were confirmed by a review of medical records with permission of the next of kin.

### Statistical Analysis

Participants were categorized in quintiles of fiber, fruits, vegetables, legumes, or whole grain consumption. We used the residuals method to adjust the assessed foods for total energy intake.

Baseline characteristics are presented according to quintiles of baseline consumption of fruit and vegetables, as mean (SD) for quantitative traits, and percentage for categorical variables.

We used Cox regression models to assess the relationship between quintiles of baseline fiber, fruit, vegetables, legumes, or whole grains consumption and the subsequent incidence of CVD during follow-up. Hazard ratios (HRs) and their 95% confidence intervals (95% CIs) were calculated using the first quintile as the reference category. We used age as the underlying time variable. Entry time was defined as the date of completion of the first questionnaire and exit time as the date of CVD, the date of the completion of the last questionnaire, or date of death, whichever occurred first.

In addition, we analyzed specific subgroups of fruit and vegetables. The definition of composite groups was based on a report by Joshipura et al. [32] which we adapted to our Food Frequency Questionnaire (FFQ) and to the usual habits of the Spanish population. The fruits and vegetables included in each subgroup are presented in Table 1.

Three multivariable models were constructed to adjust for the known or suspected predictors of CVD. First, we adjusted only for sex. In the second model, we adjusted additionally for marital status (married, single, widowed, separated, others), highest attained educational level (five categories), family history of CHD (dichotomous), type 2 diabetes (dichotomous), high blood pressure (dichotomous), dyslipidemia (dichotomous), smoking status (four categories), total energy intake (kcal/day), body mass index (kg/m^2^), alcohol intake (three categories), and use of cholesterol-lowering drugs. We also fitted a third model with additional adjustments for nutritional variables: olive oil (g/day) for all the groups and vegetables (g/day), legumes (g/day), and whole grains (g/day) in the analysis of fruit consumption; fruits (g/day), legumes (g/day), and whole grains (g/day) in an analysis that assessed vegetable consumption; fruits (g/day), vegetables (g/day), and whole grains (g/day) consumption in the analysis of legumes and fruits (g/day), vegetables (g/day), and legumes (g/day) consumption in the analysis that assessed whole grains consumption. All the models were also stratified by age in decades.

Tests for linear trend were conducted by assigning the quintile specific median value of consumption of each food item or nutrient and regressing the risk of CVD on the resulting variable, which was treated as a continuous variable.

For some foods for which the point estimates obtained with the quintiles suggested a threshold effect, we generated a new dichotomous variable and assessed the relationship between this new variable and CVD with the same multivariable models.

Statistical tests were two-sided, and *p* values of less than 0.05 were considered to indicate statistical significance.

## 3. Results

The final sample comprised 17,007 participants, of whom 61% were women and the mean age was 38 years. The median follow-up was 10.3 years (mean = 9.6 years). We observed 112 confirmed cases of CVD during this follow-up period (56 myocardial infarctions, 32 strokes, and 33 cardiovascular deaths). The mean consumption of fruits, vegetables, legumes, and whole grains was 343 g/day, 525 g/day, 23 g/day, and 12 g/day, respectively. The most consumed fruit was oranges and the most consumed vegetables were tomatoes and lettuce. The main sources of fiber were mostly vegetables (10.4 g/day) and fruits (6 g/day).

Table 2 presents the baseline characteristics of the study participants according to quintiles of baseline fiber consumption. Participants in the highest quintile were older, and more likely to be diagnosed with dyslipidemia, type 2 diabetes, and high blood pressure. As expected, participants in the higher quintiles consumed more fruits, vegetables, legumes, and whole grains, and of all the items of the fruits and vegetables groups. Also, participants with the highest fiber intake showed a higher adherence to the traditional Mediterranean diet.

When we compared the highest quintile of fruit or whole grains consumption with the lowest quintile, we observed an inverse association between them and CVD in the fully adjusted model, and also a significant inverse trend for fiber intake. Table 3 shows the association of fiber intake, fruits, vegetables, legumes, or whole grains consumption at baseline with the incidence of CVD. When we compared the participants who consumed at least 160 g/day of fruit (two servings) with those with a lower consumption we found a HR of CVD of 0.62 (95% CI 0.39–0.99, *p* = 0.045) and HR of 0.58 (95% CI 0.37–0.90; *p* = 0.015) for the participants who consumed at least 175 g/day compared with those with a lower consumption. We observed no threshold for vegetables consumption.

When we analyzed fiber intake according to its source as exposure, participants in the highest quintile of fruit fiber consumption had the lowest risk of CVD, which was significantly lower than the risk of those in the first quintile (HR 0.52, 95% CI 0.28–0.97). No statistically significant differences were found for other fiber sources.

We found no significant association between legume consumption and CVD.

When we analyzed specific groups of fruits and vegetables, only higher intakes of cruciferous vegetables were associated with CVD with a HR of 0.52 (95% CI 0.30–0.89, *p* = 0.16) for the participants who ate at least one serving/week in comparison with those who did not. We also observed an inverse significant trend in the association between cruciferous vegetable consumption and CVD (Table 4).

## 4. Discussion

In this young, highly educated, Mediterranean cohort, we found an inverse association between fiber intake and CVD. This observed association was consistent with previous studies [3,33,34], but the results for fruit and vegetables subtypes, for the different fiber sources, or for rich-fiber foods remain unclear.

We also found an inverse association between fruit consumption and fruit fiber and CVD. Our findings are consistent with various meta-analyses [12,33,34,35,36,37], even though the individual studies seldom reached statistical significance. In a previous work with participants of an old Mediterranean cohort [11], our results suggested that higher fruit consumption was likely to be inversely associated with the future occurrence of CVD, but the results were no longer statistically significant in the multivariable analysis. The EPIC study (European Prospective Investigation into Cancer and Nutrition) that included participants from several Mediterranean countries did not find an association between fruit consumption and cardiovascular mortality either [38]. A meta-analysis [33] found a marginally significant inverse association for each 4 g/day greater intake of fiber fruit with CVD (HR 0.96 (95% CI 0.93–1.00)), but another meta-analysis that included one further publication [3] found a clearly decreased risk of 8% (95% CI 2%–14%) for CHD. None of these meta-analyses included studies with Mediterranean populations. In our work, we also found that consumptions of at least two portions a day of fruit (160 g/day) could be protective against CVD, and higher consumptions did not appear to provide additional protection.

We found no significant association with vegetable consumption nor with fiber from vegetables. Similarly, a recent meta-analysis [3] did not find a statistically significant association between vegetable fiber and CVD. It is also noteworthy that in our study vegetable consumption was very high (mean 525 g/day) and the median of the first quintile was already 209 g/day. Lack of variability in a lower range of vegetable consumption may have precluded us from finding significant associations. In addition, the number of observed cases was low, as expected in this educated, slim, and young Mediterranean cohort. The statistical power of our study would be sufficient only to detect relative risks between extreme quintiles in the range of 0.40–0.55, but not other relative risks closer to the null. However, we expected to find strong associations, on the basis of a previous case-control study conducted by our group [39].

Fruit and vegetables are rich sources of fiber, but they also provide antioxidants, vitamin K, folate, and other phytochemicals—some of them have other micronutrients, thus leading to heterogeneity between the different types. Therefore, some classifications have been suggested according these differences. Nevertheless, studies that have assessed the association between a food or group and CVD are scarce. Oude Griep et al. [40] found no significant association between different varieties of fruits and vegetables and CHD or stroke, but found a strong correlation between the different fruit varieties and total fruit intake (0.72), and a less strong correlation between the different vegetables varieties and total vegetable consumption (0.53). Their results suggest that the quantity of fruit and vegetable consumption may be more relevant than their variety for the prevention of CVD. In a different classification according to the color of vegetables, the same authors [41,42] found a significant protective association with stroke only for white fruits and vegetables; no significant association was observed for CHD. In our analysis, we found an inverse association between cruciferous vegetable consumption and CVD, where eating at least one serving of cruciferous vegetables per week could reduce the risk of CVD by 48%. Our results differ from other studies that found no association between cruciferous vegetables and stroke [12] or CHD [32,43], but are consistent with our results from a previous study on an old Mediterranean cohort [11]. Bendinelli [9] analyzed the association between fruit and vegetable consumption and CHD in a Mediterranean cohort of women, and found an inverse association with green leafy vegetables, which was consistent with other studies [32,44,45], but did not report the results for cruciferous vegetables alone. Cruciferous vegetables contain sulforaphane [7], the most widely studied and best characterized isothiocyanate [46] which is a long-acting antioxidant, but also has anti-inflammatory activity by inhibition of cytokine production. The relationship between sulforaphane and the atherosclerosis process seems to be clear [46]. In animal models, sulforaphane has shown an inverse association with hypertension, diabetes, and diabetes complications [47,48].

A higher consumption of legumes did not have any association with the risk of CVD in our cohort. Legumes are high in vegetable proteins and dietary fiber, and have a low glycemic index. To our knowledge, only Bazzano et al. [49] and Wang et al. [50] reported an inverse association between the consumption of legumes and CVD, with tangible benefits observed for the consumption of legumes at least four times per week. A recently published meta-analysis [51] found an inverse association between legume consumption and CHD (HR 0.90 CI 95% 0.84–0.97). Additionally, the authors of this latter meta-analysis also found a marginally significant association between legume consumption and CVD (HR 0.94 CI 95% 0.89–1.00). Our participants had a high legume consumption (median in the fifth quintile: 36 g/day) and a priori we expected to find a risk reduction. Legumes are an important food in the Mediterranean diet and Trichopoulou et al. [13] found that the observed inverse association between adherence to the Mediterranean diet and cardiovascular mortality was toned down when legumes were taken out from their score. Another meta-analysis [52] conducted with 20 studies that analyzed the association between adherence to the Mediterranean diet or its individual components and CVD also found an inverse association between legume consumption and CVD (HR 0.90 CI 95% 0.83–0.98). Our results are similar in the magnitude and the direction of the association, but we did not achieve statistical significance. Again, the low number of cases could explain this result. Moreover, there are important differences in composition across legumes in macronutrients [53] (specifically in their protein content), fiber content, micronutrients, and phytochemicals, which could also explain the differences across different cohorts. Additionally, there are differences in the way they are cooked in the different Mediterranean countries, which could also contribute to the diverse results in the different studies.

We found an inverse association between whole grain consumption and risk of CVD (HR for the fifth vs. first quintile of 0.43 (95% CI 0.20–0.93)). Our study is consistent with a meta-analysis of ten cohort studies [21] that found an inverse association between whole grain consumption and risk of CHD, stroke, and CVD, with no evidence of linearity and with a stronger risk reduction from no consumption up to 50 g/day than with higher intakes. Most of the studies included in the last meta-analysis were from USA, only one of the included cohorts originating from a Mediterranean setting, and even then, only cardiovascular mortality was assessed. We found no association between whole grain consumption and CVD in a cohort with older Mediterranean participants [11].

Different plant foods, including fruits, vegetables, and whole grains among others, have their distinctive phytochemical contents. These distinctive phytochemicals have different biochemical properties, such as molecular size and solubility [5]. All these properties will affect the interactions between the various biochemicals and thus their antioxidant or anti-inflammatory effect. Also, different plant-based foods contain different fiber types. Eating a wide variety of fruits, vegetables, whole grains, and other plant foods and following a health dietary pattern—like the Mediterranean diet—has been postulated as being more important for CVD prevention than eating isolated food items [54]. A recent meta-analysis has shed light on this association [52], finding that some of the individual components did not achieve significance, but that the overall diet decreased CVD disease rates.

We acknowledge that our study may have some limitations. First, our participants have a high educational level, and are young and predominantly females, potentially limiting the generalizability of our results. However, the high educational level of our participants enhances the quality of their self-reported information and increases the internal validity of our results. In addition, lack of representativeness does not preclude from finding associations valid for other populations [55]. In fact, there are no biological mechanisms that suggest that our observed associations may no longer hold for other populations. Finally, literature on the covered topic in middle-aged cohort was previously absent. Second, the use of a validated FFQ does not rule out the existence of an information bias, but it has good or reasonable correlation coefficients for the considered exposures. Since the possible measurement error in the assessment of diet is expected to be non-differential, it would presumably bias our results towards the null value. Third, the absence of repeated measurements may have also led to a misclassification bias, because our participants may have experienced changes in their consumption along the follow-up. Fourth, we might have missed some CVD events. Nevertheless, all of our participants are university graduates, who are highly educated and highly motivated, and more than half of them are health professionals, thereby reducing the risk of underreporting. Additionally, we regularly consulted the National Death Index. Fifth, the incidence of CVD was low in our cohort. Thus, we may have low statistical power to assess associations, especially in adjusted models. Nevertheless, it is noteworthy that the point estimates hardly changed with successive adjustment and that the point estimates point towards the a priori expected association based on the available literature. Finally, observing significant associations may have been hindered by a considerably high consumption of healthy foods in the lowest quintiles, compared to other available studies. High consumption of the considered food items may show protective effects when compared to really low consumptions, but may show no additional protection beyond moderate consumptions.

The major strengths of the current study include its prospective design, its relatively large sample and long-term follow-up, and its high retention rate. Another potential strength is the use of a validated FFQ, with good correlations with the analyzed foods. Moreover, we adjusted the models for a wide array of potential confounders, in an attempt to control for potential confounders.

In conclusion, consumptions of at least two servings/day of fruit, and one serving/week of cruciferous vegetables or whole grains may be protective against CVD in young Mediterranean populations.

## Figures and Tables

**Table 1:** Definitions for various fruit and vegetable subgroups.

Subgroups	Individual Foods
Orange group	Orange, tangerine, grapefruit
Apple/pear group	Apple/pear
Green leafy vegetables	Spinach, lettuce, chard greens
Cruciferous vegetables	Broccoli, cabbage, cauliflower, Brussels sprouts
β-carotene-rich fruits and vegetables	Carrots, spinach, pumpkin, chard greens
Lutein-rich fruits and vegetables	Spinach, chard greens
Lycopene-rich fruits and vegetables	Tomatoes, tomato sauce, “gazpacho” *
Vitamin C-rich fruits and vegetables	Orange, tangerine, grapefruit, peppers

* Cold tomato-based soup with pepper, cucumber, garlic, and onion.

**Table 2:** Baseline characteristics * of the study participants according to baseline quintiles of fiber intake, SUN (Seguimiento Universidad de Navarra, Follow-up) Project 1999–2013.

	Quintiles of Fiber Intake				
	Q1	Q2	Q3	Q4	Q5
Age (years)	34 (10)	36 (11)	38 (12)	40 (12)	42 (13)
Sex (% women)	60.6	60.6	60.6	60.6	60.6
Marital Status, %					
Single	55.3	47.4	42.9	40.6	37.9
Married	41.8	48.9	52.6	53.8	55.6
Others	2.9	3.8	4.6	5.6	6.5
Highest attained educational level					
Degree or licenciate degree, %	56	53	53	52	53
Master’s degree, %	7	8	8	8	8
Doctoral degree, %	7	9	11	11	11
Family history of CHD, %	11	12	14	14	16
Use of lipid-lowering drugs, %	1.2	1.5	3.0	3.1	4.0
Type 2 diabetes, %	0.7	0.9	1.7	2.2	3.0
Body mass index (Kg/m^2^)	23 (11)	23 (3.3)	24 (3.5)	24 (3.5)	24 (3.5)
Hypertension, %	3.7	4.9	6.7	7.9	9.3
Dyslipidemia, %	11.7	14.9	16.9	16.2	20.1
Smoking status, %					
Current smoker	28.5	23.4	21.6	18.6	16.1
Former smoker	22.5	26.5	28.0	32.6	33.3
Carbohydrate intake (% of total energy)	40 (7)	42 (7)	43 (7)	44 (7)	48 (7)
Protein intake (% of total energy)	17 (4)	18 (3)	18 (3)	19 (3)	19 (3)
Fat intake (% of total energy)	40 (7)	38 (6)	37 (6)	36 (6)	32 (6)
Olive oil consumption (g/day) **	18 (14)	18 (14)	19 (13)	19 (13)	18 (13)
Fiber intake (g/day) **	16 (3.4)	22 (1.5)	26 (1.6)	31 (2.1)	44 (10.0)
Fruit consumption (g/day) **	144 (116)	246 (143)	312 (159)	407 (219)	604 (419)
Vegetable consumption (g/day) **	271 (138)	397 (151)	493 (176)	601 (218)	863 (458)
Legume consumption (g/day) **	16 (9)	20 (10)	22 (12)	24 (14)	31 (29)
Whole grains (g/day) **	2 (10)	6 (15)	10 (21)	16 (29)	32 (52)
Orange group (portions/week) **	1.8 (2.7)	3.3 (3.5)	4.5 (4.4)	5.7 (5.3)	8.2 (7.7)
Apples/pear (portions/week) **	1.1 (1.5)	1.9 (2.0)	2.5 (2.6)	3.3 (3.4)	5.2 (5.8)
Lutein-rich fruits and vegetables (portions/week) **	0.4 (0.4)	0.6 (0.6)	0.8 (0.7)	1.0 (1.0)	1.7 (1.9)
Cruciferous (portions/week) **	0.3 (0.4)	0.5 (0.5)	0.6 (0.6)	0.8 (0.9)	1.2 (1.6)
Green-leafy vegetables (portions/week) **	2.5 (1.9)	3.6 (2.3)	4.5 (2.5)	5.5 (3.1)	7.8 (5.6)
Lycopene-rich fruits and vegetables (portions/week) **	2.0 (2.1)	3.7 (2.3)	3.7 (2.8)	4.5 (3.1)	6.2 (5.5)
Carotene-rich fruits and vegetables (portions/week) **	1.0 (1.0)	1.7 (1.2)	2.3 (1.5)	3.0 (1.9)	5.1 (4.1)
Vitamin C-rich fruits and vegetables (portions/week) **	3.1 (3.0)	5.0 (3.8)	6.5 (4.6)	8.1 (5.6)	11.5 (8.7)
Alcohol consumption (g/day) **	6.1 (11.7)	5.0 (7.8)	4.3 (6.8)	4.2 (12.9)	3.5 (6.0)
Moderate alcohol consumption (%) **	24.6	22.38	19.43	18.32	15.08
Physical activity (METs-h/week) **	21.6 (19.0)	21.6 (19.0)	22.4 (19.4)	23.3 (20.0)	24.3 (20.0)
Total energy intake (Kcal/day) **	2525 (616)	2283 (591)	2223 (595)	2237 (610)	2420 (610)
MDS (0–9 items) ***	2.7 (1.4)	3.5 (1.5)	4.1 (1.4)	4.8 (1.4)	5.6 (1.4)

* Mean (SD) unless otherwise stated; ** Adjusted for total energy intake by the residual method; *** MDS, Mediterranean diet score [13]; CHD, coronary heart disease; METs, metabolic equivalent of task.

**Table 3:** Hazard ratios (HRs) (95% confidence intervals) of cardiovascular disease (CVD) according to baseline quintiles (Q) of fiber, fruit, vegetables, pulses, or whole-grain consumption.

**Quintiles of Fiber Intake**						
	**Q1**	**Q2**	**Q3**	**Q4**	**Q5**	***p* for Trend**
*N*	3402	3401	3402	3401	3401	
Time at risk	33,643	33,107	32,496	32,132	31,381	
# of cases	18	24	22	22	26	
Median g/day	16.76	22.06	26.09	31.12	40.88	
Sex adjusted	1 (ref)	1.24 (0.66–2.30)	0.77 (0.41–1.45)	0.67 (0.36–1.28)	0.57 (0.31–1.06)	0.015
Multivariable adjusted 1	1 (ref)	1.31 (0.70–2.49)	0.81 (0.42–1.57)	0.70 (0.37–1.35)	0.61 (0.32–1.16)	0.025
Multivariable adjusted 2	1 (ref)	1.32 (0.70–2.49)	0.81 (0.42–1.56)	0.70 (0.36–1.35)	0.60 (0.32–1.15)	0.024
**Quintiles of Fruit Consumption**						
	**Q1**	**Q2**	**Q3**	**Q4**	**Q5**	***p* for Trend**
*N*	3402	3401	3402	3401	3401	
Time at risk	33,824	32,929	32,684	31,714	31,608	
# of cases	23	19	17	25	28	
Median g/day	90	192	280	398	653	
Sex adjusted	1 (ref)	0.60 (0.32–1.11)	0.45 (0.24–0.85)	0.49 (0.28–0.88)	0.45 (0.25–0.81)	0.036
Multivariable adjusted 1	1 (ref)	0.61 (0.32–1.17)	0.48 (0.24–0.94)	0.51 (0.27–0.94)	0.48 (0.26–0.87)	0.057
Multivariable adjusted 3	1 (ref)	0.62 (0.32–1.18)	0.50 (0.26–0.98)	0.54 (0.29–1.00)	0.51 (0.27–0.95)	0.114
**Quintiles of Vegetable Consumption**						
	**Q1**	**Q2**	**Q3**	**Q4**	**Q5**	***p* for Trend**
*N*	3402	3401	3402	3401	3401	
Time at risk	33,695	32,816	32,870	32,100	31,278	
# of cases	20	24	25	18	25	
Median g/day	209	348	468	609	884	
Sex adjusted	1 (ref)	1.18 (0.65–2.15)	1.06 (0.58–1.92)	0.71 (0.37–1.35)	0.80 (0.44–1.47)	0.191
Multivariable adjusted 1	1 (ref)	1.29 (0.70–2.38)	1.07 (0.58–1.96)	0.77 (0.40–1.48)	0.81 (0.44–1.49)	0.180
Multivariable adjusted 4	1 (ref)	1.37 (0.74–2.54)	1.14 (0.62–2.11)	0.86 (0.44–1.67)	0.96 (0.51–1.82)	0.458
**Quintiles of Legumes Consumption**						
	**Q1**	**Q2**	**Q3**	**Q4**	**Q5**	***p* for Trend**
*N*	3402	3401	3402	3401	3401	
Time at risk	32,311	32,682	32,726	33,244	31,796	
# of cases	26	24	20	15	27	
Median g/day	8.4	15.2	20	25	36	
Sex adjusted	1 (ref)	0.91 (0.52–1.60)	0.78 (0.43–1.40)	0.58 (0.30–1.10)	0.96 (0.59–1.65)	0.674
Multivariable adjusted 1	1 (ref)	0.92 (0.52–1.62)	0.80 (0.44–1.45)	0.58 (0.30–1.11)	0.92 (0.53–1.59)	0.559
Multivariable adjusted 5	1 (ref)	0.95 (0.54–1.68)	0.83 (0.46–1.52)	0.59 (0.31–1.15)	0.95 (0.54–1.66)	0.622
**Quintiles of Whole Grains Consumption**						
	**Q1**	**Q2**	**Q3**	**Q4**	**Q5**	***p* for Trend**
*N*	3402	3401	3402	3401	3401	
Time at risk	34,089	34,090	33,207	32,620	31,255	
# of cases	26	17	25	28	16	
Median g/day	0	0	1.9	5.7	54.7	
Sex adjusted	1 (ref)	0.59 (0.31–1.10)	0.79 (0.45–1.37)	0.71 (0.41–1.22)	0.51 (0.27–0.95)	0.110
Multivariable adjusted 1	1 (ref)	0.49 (0.24–0.99)	0.62 (0.29–1.31)	0.48 (0.20–1.15)	0.40 (0.19–0.86)	0.102
Multivariable adjusted 6	1 (ref)	0.51 (0.25–1.02)	0.64 (0.30–1.36)	0.49 (0.20–1.18)	0.43 (0.20–0.93)	0.149

Note: Multivariable adjusted 1: sex, marital status (five categories), highest attained educational level (five categories), family history of CHD (yes/no), type 2 diabetes (yes/no), high blood pressure (yes/no), dyslipidaemia (yes/no), smoking status (four categories), total energy intake (kcal/day), body mass index, physical activity (METs-h/day), alcohol intake (three categories) and use of lipid-lowering drugs, and stratified by age in decades. Age as underlying time variable. Multivariable adjusted 2: additionally adjusted for and olive oil consumption (g/day). Multivariable adjusted 3: additionally adjusted for olive oil (g/day), vegetables, legumes (g/day), and whole-grains (g/day) consumption. Multivariable adjusted 4: additionally adjusted for olive oil (g/day), fruits (g/day), legumes (g/day), and whole-grains (g/day) consumption. Multivariable adjusted 5: additionally adjusted for olive oil (g/day), fruits (g/day), vegetables (g/day), and whole-grains (g/day) consumption. Multivariable adjusted 6: additionally adjusted for olive oil (g/day), fruits (g/day), vegetables (g/day), and legumes (g/day) consumption.

**Table 4:** Hazard ratios (HRs) of CVD according to baseline quintiles (Q) of specific groups of fruits and vegetables consumption. (HR and 95% confidence intervals).

**Orange Group**						
	**Q1**	**Q2**	**Q3**	**Q4**	**Q5**	***p* for Trend**
*N*	3402	3401	3402	3401	3401	
Time at risk	32,607	32,155	32,253	32,686	33,057	
# of cases	21	22	21	21	27	
Median (servings/week)	0.23	1.58	3.09	5.64	15.34	
Sex adjusted	1 (ref)	0.80 (0.43–1.46)	0.73 (0.40–1.34)	0.53 (0.29–0.98)	0.60 (0.34–1.08)	0.166
Multivariable adjusted 1	1 (ref)	0.81 (0.43–1.55)	0.79 (0.42–1.50)	0.56 (0.30–1.05)	0.65 (0.35–1.19)	0.234
Multivariable adjusted 2	1 (ref)	0.81 (0.43–1.56)	0.80 (0.42–1.52)	0.56 (0.30–1.07)	0.65 (0.35–1.20)	0.267
**Apple/Pear Group**						
	**Q1**	**Q2**	**Q3**	**Q4**	**Q5**	***p* for Trend**
*N*	3402	3401	3402	3401	3401	
Time at risk	33,274	32,993	32,058	31,793	32,641	
# of cases	24	16	17	25	30	
Median (servings/week)	0.16	0.79	1.55	3.12	6.68	
Sex adjusted	1 (ref)	0.51 (0.27–0.97)	0.47 (0.25–0.89)	0.75 (0.42–1.32)	0.61 (0.35–1.06)	0.715
Multivariable adjusted 1	1 (ref)	0.46 (0.23–0.92)	0.44 (0.22–0.88)	0.74 (0.41–1.35)	0.61 (0.34–1.08)	0.816
Multivariable adjusted 2	1 (ref)	0.47 (0.24–0.93)	0.45 (0.23–0.91)	0.79 (0.44–1.44)	0.65 (0.37–1.16)	0.997
**Green Leafy Group**						
	**Q1**	**Q2**	**Q3**	**Q4**	**Q5**	***p* for Trend**
*N*	3402	3401	3402	3401	3401	
Time at risk	32,830	32,494	32,226	32,498	32,711	
# of cases	19	23	25	22	23	
Median (servings/week)	1.14	2.81	3.99	6.16	8.21	
Sex adjusted	1 (ref)	1.47 (0.79–2.72)	1.42 (0.77–2.59)	0.90 (0.48–1.66)	0.92 (0.50–1.71)	0.268
Multivariable adjusted 1	1 (ref)	1.48 (0.79–2.77)	1.57 (0.85–2.91)	0.90 (0.48–1.68)	0.97 (0.52–1.80)	0.298
Multivariable adjusted 2	1 (ref)	1.54 (0.82–2.89)	1.61 (0.87–2.99)	0.93 (0.49–1.75)	1.05 (0.56–1.96)	0.431
**Cruciferous Group**						
	**Q1**	**Q2**	**Q3**	**Q4**	**Q5**	***p* for Trend**
*N*	3402	3401	3402	3401	3401	
Time at risk	32,604	32,972	32,488	32,817	31,878	
# of cases	19	26	27	21	19	
Median (servings/week)	0	0.37	0.48	0.93	1.07	
Sex adjusted	1 (ref)	1.03 (0.56–1.88)	1.25 (0.69–2.27)	0.81 (0.43–1.51)	0.56 (0.30–1.08)	0.035
Multivariable adjusted 1	1 (ref)	0.97 (0.52–1.79)	1.35 (0.73–2.47)	0.82 (0.43–1.55)	0.54 (0.28–1.04)	0.033
Multivariable adjusted 2	1 (ref)	0.98 (0.53–1.81)	1.37 (0.75–2.52)	0.83 (0.44–1.58)	0.56 (0.29–1.09)	0.046
**Carotene-Rich Fruits and Vegetables**						
	**Q1**	**Q2**	**Q3**	**Q4**	**Q5**	***p* for Trend**
*N*	3402	3401	3402	3401	3401	
Time at risk	33,385	33,226	32,328	32,002	31,817	
# of cases	25	19	24	20	24	
Median (servings/week)	0.44	1.16	1.78	3–28	5.92	
Sex adjusted	1 (ref)	0.69 (0.38–1.26)	0.82 (0.47–1.45)	0.62 (0.34–1.14)	0.60 (0.33–1.06)	0.111
Multivariable adjusted 1	1 (ref)	0.74 (0.40–1.38)	0.90 (0.50–1.62)	0.67 (0.36–1.24)	0.64 (0.35–1.14)	0.142
Multivariable adjusted 2	1 (ref)	0.77 (0.41–1.43)	0.92 (0.51–1.67)	0.71 (0.38–1.31)	0.68 (0.38–1.23)	0.218
**Lutein-Rich Fruits and Vegetables**						
	**Q1**	**Q2**	**Q3**	**Q4**	**Q5**	***p* for Trend**
*N*	3402	3401	3402	3401	3401	
Time at risk	32,106	32,994	32,316	32,995	32,408	
# of cases	21	23	22	18	27	
Median (servings/week)	0	0.4	0.58	0.98	2.88	
Sex adjusted	1 (ref)	0.90 (0.49–1.65)	0.90 (0.49–1.65)	0.76 (0.40–1.43)	0.76 (0.43–1.36)	0.381
Multivariable adjusted 1	1 (ref)	1.07 (0.58–1.99)	1.0 (0.54–1.87)	0.82 (0.43–1.56)	0.81 (0.45–1.48)	0.354
Multivariable adjusted 2	1 (ref)	1.10 (0.59–2.05)	1.03 (0.56–1.93)	0.84 (0.44–1.60)	0.86 (0.47–1.55)	0.439
**Lycopene-Rich Fruits and Vegetables**						
	**Q1**	**Q2**	**Q3**	**Q4**	**Q5**	***p* for Trend**
*N*	3402	3401	3402	3401	3401	
Time at risk	34,332	33,347	32,602	31,537	31,041	
# of cases	23	26	19	23	21	
Median (servings/week)	0.44	1.91	3.24	4.87	7.23	
Sex adjusted	1 (ref)	1.04 (0.59–1.84)	0.81 (0.44–1.50)	0.78 (0.43–1.39)	0.70 (0.38–1.27)	0.137
Multivariable adjusted 1	1 (ref)	0.98 (0.54–1.76)	0.80 (0.43–1.49)	0.81 (0.44–1.47)	0.69 (0.38–1.28)	0.181
Multivariable adjusted 2	1 (ref)	1.0 (0.55–1.79)	0.8 (0.43–1.49)	0.84 (0.46–1.53)	0.73 (0.40–1.35)	0.249
**Vitamin C-rich fruits and vegetables**						
	**Q1**	**Q2**	**Q3**	**Q3**	**Q5**	***p* for Trend**
*N*	3402	3401	3402	3401	3401	
Time at risk	32,746	32,672	32,371	32,628	32,341	
# of cases	20	21	25	19	27	
Median (servings/week)	1.46	3.4	5.33	7.84	16.41	
Sex adjusted	1 (ref)	0.78 (0.42–1.46)	0.91 (0.50–1.65)	0.55 (0.29–1.03)	0.61 (0.33–1.11)	0.103
Multivariable adjusted 1	1 (ref)	0.79 (0.42–1.53)	0.99 (0.54–1.84)	0.56 (0.29–1.07)	0.66 (0.38–1.22)	0.171
Multivariable adjusted 2	1 (ref)	0.83 (0.43–1.61)	1.00 (0.54–1.86)	0.58 (0.30–1.11)	0.68 (0.37–1.27)	0.191

Note: Multivariable adjusted 1: sex, marital status (five categories), studies (five categories), family history of CHD (yes/no), type 2 diabetes (yes/no), high blood pressure (yes/no), dyslipidemia (yes/no), smoking status (four categories), total energy intake (kc/day), body mass index, physical activity (METs-h/day), alcohol intake (three categories), stratified by age in decades. Age as underlying time variable. Multivariable adjusted 2: additionally adjusted for legumes (g/day), whole-grains (g/day), and olive oil consumption (g/day).
